# A practical approach on the classifications of myeloid neoplasms and acute leukemia: WHO and ICC

**DOI:** 10.1186/s13045-024-01571-4

**Published:** 2024-07-29

**Authors:** Wenbin Xiao, Valentina Nardi, Eytan Stein, Robert P. Hasserjian

**Affiliations:** 1 Department of Pathology and Laboratory Medicine, Memorial Sloan Kettering Cancer Center, New York, NY USA; 2 Department of Pathology, Mass General Brigham, Harvard Medical School, Boston, MA USA; 3 Department of Medicine, Memorial Sloan Kettering Cancer Center, New York, NY USA

## Abstract

In 2022, two new classifications of myeloid neoplasms and acute leukemias were published: the 5th edition WHO Classification (WHO-HAEM5) and the International Consensus Classification (ICC). As with prior classifications, the WHO-HAEM5 and ICC made updates to the prior classification (revised 4th edition WHO Classification, WHO-HAEM4R) based on a consensus of groups of experts, who examined new evidence. Both WHO-HAEM5 and ICC introduced several new disease entities that are based predominantly on genetic features, superseding prior morphologic definitions. While it is encouraging that two groups independently came to similar conclusions in updating the classification of myeloid neoplasms and acute leukemias, there are several divergences in how WHO-HAEM5 and ICC define specific entities as well as differences in nomenclature of certain diseases. In this review, we highlight the similarities and differences between the WHO-HAEM5 and ICC handling of myeloid neoplasms and acute leukemias and present a practical approach to diagnosing and classifying these diseases in this current era of two divergent classification guidelines.

## Introduction

The 3rd edition WHO Classification of hematopoietic neoplasms (WHO-HAEM3) published in 2001 was the first comprehensive classification system of myeloid neoplasms and acute leukemias. The WHO-HAEM3 included aspects of the French-American-British classification of MDS and AML [1], but also applied principles developed in the Revised European-American Classification of Lymphoid Neoplasms (REAL) classification [2], i.e. that a combination of morphology, immunophenotype, genetic features, and clinical features defines disease entities [3]. For example, while most MDS disease subtypes were defined purely by morphologic features (the percentage of blasts in bone marrow and blood, the degree of morphologic dysplasia, and ring sideroblasts), MDS associated with isolated del(5q) was defined mainly on a cytogenetic feature. In AML, there were 4 disease subtypes defined by cytogenetic translocations, with the remainder defined based on morphology and clinical features. The 4th edition (WHO-HAEM4) and revised 4th edition (WHO-HAEM4R) classifications, published in 2008 and 2016 respectively [4, 5], made relatively minor changes in the definitions and nomenclature of MDS disease subtypes, but progressively introduced more genetically-defined subtypes of AML.

In 2022, two new classifications of myeloid neoplasms and acute leukemias were published: the 5th edition WHO Classification (WHO-HAEM5) and the International Consensus Classification (ICC) [6, 7]. The reasons behind the publication of two separate classifications are reviewed elsewhere [8, 9]. As with prior classifications, the WHO-HAEM5 and ICC made updates to the prior classification (WHO-HAEM4R) based on a consensus of groups of experts, who examined new evidence. In particular, a large body of evidence has recently accumulated on the genetic pathogenesis of myeloid neoplasms and their relationship to myeloid precursor lesions. Genetic testing has also revealed new distinct subgroups that are more biologically accurate than prior morphologic markers of disease. Accordingly, both WHO-HAEM5 and ICC introduced new disease entities that are based predominantly on genetic features, superseding prior morphologic definitions. While it is encouraging that two groups independently came to similar conclusions in updating myeloid neoplasm entities, there are several divergences in how WHO-HAEM5 and ICC define specific entities. There are also several differences in nomenclature between the two classifications, which likely reflect differences in how the two groups sought to apply descriptive names to the same entity as well as the influence of the nomenclature of other disease groups. For example, while the ICC retained the term “myelodysplastic syndrome”, the WHO-HAEM5 changed the name to “myelodysplastic neoplasm” in consonance with the related entities myeloproliferative neoplasms (MPN) and myelodysplastic/myeloproliferative neoplasms (MDS/MPN). Conversely, the ICC felt that retaining the historic and traditional “syndrome” nomenclature superseded the rationale to apply a more scientifically accurate terminology of “neoplasm”. In order to avoid confusion with the commonly abbreviated MPN and MDS/MPN entities, the WHO-HAEM5 retained the “MDS” abbreviation for “myelodysplastic neoplasms”.

In this review, we highlight the similarities and differences between the WHO-HAEM5 and ICC handling of myeloid neoplasms and acute leukemias and present a practical approach to diagnosing and classifying these diseases in this current era of two divergent classification guidelines. The main categories of myeloid neoplasms and their precursor lesions, which are the same in both classifications (with minor nomenclature differences), are listed in Table 1.

**Table 1:** Summary of myeloid neoplasm entities

Group	Key feature(s)	Year introduced into WHO/ICC myeloid classifications
Myelodysplastic syndromes/neoplasms	Ineffective hematopoiesis resulting in cytopenia and morphologic dysplasia	2001 (WHO-HAEM3)
Myeloproliferative neoplasms*	Overexuberant myeloid proliferation, usually resulting in elevated blood count(s)	2001 (WHO-HAEM3)
Myelodysplastic/myeloproliferative neoplasms	Mixed features of cytopenia, morphologic dysplasia, and proliferation of one or more myeloid lineages.	2001 (WHO-HAEM3)
Acute myeloid leukemia	Impaired myeloid maturation with accumulation of myeloid blasts	2001 (WHO-HAEM3)
Acute leukemia of ambiguous lineage	Accumulation of blasts with ambiguous or mixed myeloid/lymphoid lineages.	2001 (WHO-HAEM3)
Mastocytosis	Neoplastic proliferation of mast cells	2008 (WHO-HAEM4)
Myeloid/lymphoid neoplasms with eosinophilia and tyrosine kinase gene fusions	Stem cell hematopoietic disorder associated with a genetic rearrangement activating a specific tyrosine kinase, usually associated with eosinophilia	2008 (WHO-HAEM4)
Blastic plasmacytoid dendritic cell neoplasm	Neoplastic proliferation of blastic plasmacytoid dendritic cells	2008 (WHO-HAEM4)
Myeloid neoplasms with germline predisposition	Germline mutation in gene associated with increased risk of myeloid malignancy	2016 (WHO-HAEM4R)
Myeloid neoplasm precursor lesions	Clonal myeloid proliferation without morphologic features of malignancy	2022 (WHO-HAEM5 and ICC)

* The terminology of “chronic myeloproliferative diseases” was used in WHO-HAEM3 and it was renamed “myeloproliferative neoplasms” in WHO-HAEM4

### Myeloid neoplasm precursor lesions

**Fig. 1:**
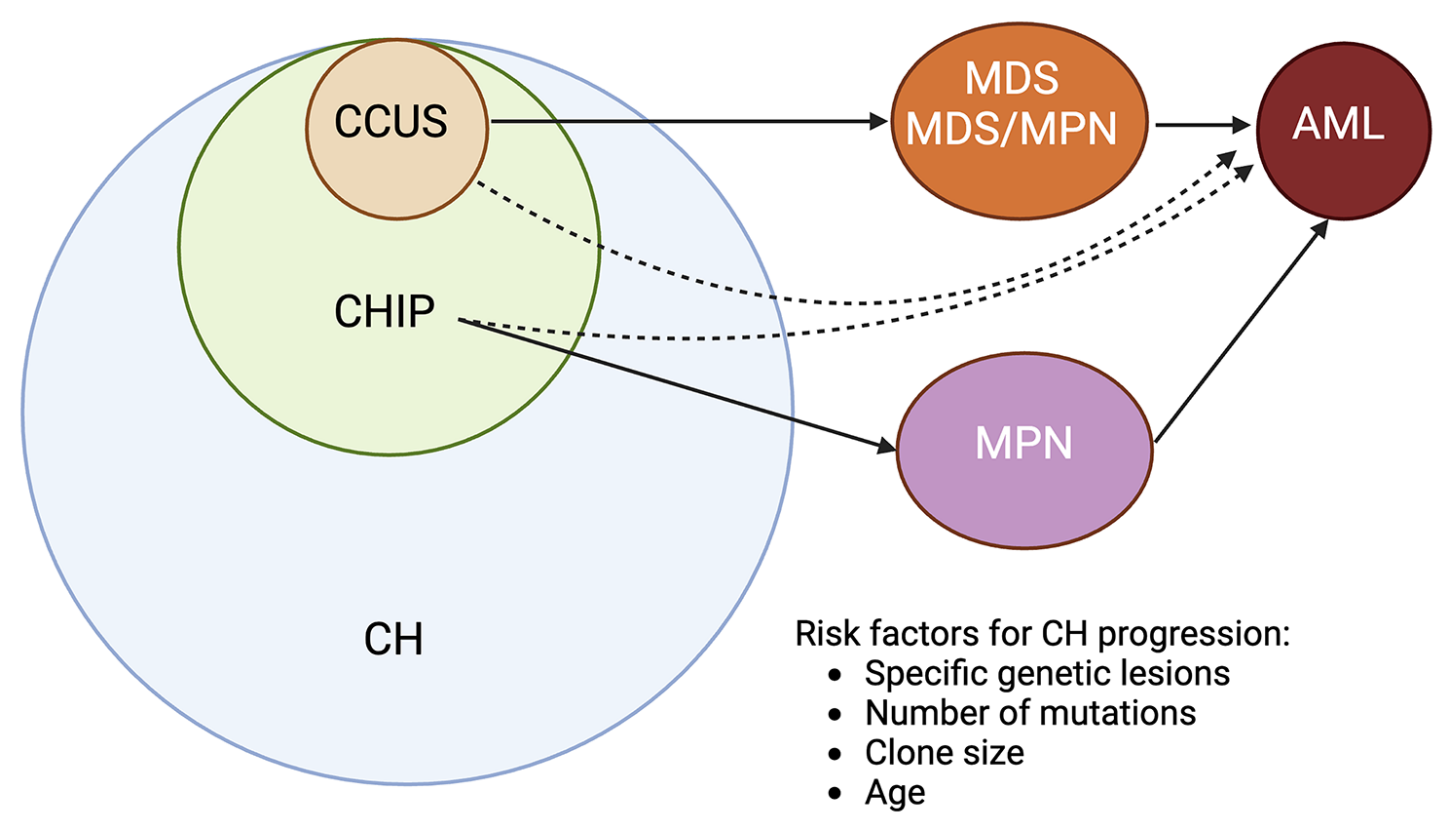
Precursor lesions and their evolution to myeloid neoplasm. Other clonal proliferations with cytopenia such as VEXAS syndrome, PNH and aplastic anemia are not shown here. CH, clonal hematopoiesis. CHIP, clonal hematopoiesis of indeterminate potential. CCUS, clonal cytopenia of undetermined significance. MDS, myelodysplastic neoplasms/syndromes. MPN, myeloproliferative neoplasms. AML, acute myeloid leukemia

Clonal hematopoiesis (CH) is a myeloid neoplasm precursor lesion characterized by overrepresentation of blood cells derived from a single clone, identified by its somatic mutations, cytogenetic aberrations, and/or copy number abnormalities detected on genetic testing [10, 11]. Clonal hematopoiesis of indeterminate potential (CHIP) refers to CH specifically harboring either a somatic mutation in a myeloid neoplasm driver gene with a variant allele frequency (VAF) of at least 2% or a non–MDS-defining clonal cytogenetic aberration, in a patient lacking a hematologic neoplasm or unexplained cytopenia [12] (Table 2). Clonal cytopenia of undetermined significance (CCUS) is defined as CHIP detected in the presence of one or more persistent unexplained cytopenias, while diagnostic criteria for any defined myeloid neoplasm are not met. Both WHO-HAEM5 and ICC for the first time included CHIP and CCUS as myeloid precursor lesions. The ICC also recognized VEXAS syndrome and paroxysmal nocturnal hemoglobinuria (PNH), both caused by somatic mutations, as clonal myeloid proliferations associated with cytopenia that are not equivalent to MDS unless diagnostic morphologic criteria for MDS are met. Some individuals with myeloid neoplasm precursor lesions progress to MDS or other myeloid neoplasms (Fig. 1). However, further study is warranted to better define the determinants of their progression risk [13, 14]. Moreover, refinement in the distinction between higher-risk CCUS and lower-risk MDS is warranted: these are biologically and prognostically similar and are currently separated arbitrarily by the absence versus presence of significant morphologic dysplasia, the identification of which can be subjective [15, 16].

**Table 2:** Definitions of CH, CHIP and CCUS

Criteria	CH	CHIP	CCUS
Unexplained cytopenia*	Yes or No	No	Yes, ≥4 months
and			
Mutations	Any somatic mutation(s) in hematopoietic cells	Mutation(s) in myeloid neoplasm driver gene(s) VAF≥2%	Mutation(s) in myeloid neoplasm driver gene(s) VAF≥2%**
and/or			
Cytogenetics	Acquired clonal chromosomal abnormality in hematopoietic cells	Acquired clonal chromosomal abnormality in myeloid cells	Acquired clonal cytogenetic abnormality in hematopoietic cells**
and			
Other features	No current or prior hematologic malignancy Excluded from CHIP or CCUS due to VAF < 2% or prior history of hematologic malignancy	No current or prior hematologic malignancy	No current or prior hematologic malignancy

*Abbreviations* CH, clonal hematopoiesis; CHIP, clonal hematopoiesis of indeterminate potential; CCUS, clonal cytopenia of undetermined significance

*Defined as anemia (HGB < 13 g/dL in males, < 12 g/dL in females), thrombocytopenia (platelets < 150 × 10^9^/L), and/or neutropenia (ANC < 1.8 × 10^9^/L)

**In ICC, certain MDS-defining genetic lesions are excluded and mandate a diagnosis of MDS in a cytopenic patient: multi-hit *TP53* mutation (VAF ≥10%), *SF3B1* mutation (VAF ≥10%), complex karyotype (≥3 independent aberrations, except -Y), del(5q), -7, or del(7q)

### MPN

Myeloproliferative neoplasms (MPN) include chronic myeloid leukemia (CML), the *JAK2/MPL/CALR*-associated MPN (essential thrombocythemia, primary myelofibrosis, and polycythemia vera), chronic neutrophilic leukemia (CNL), chronic eosinophilic leukemia, and MPN-NOS/unclassifiable. WHO-HAEM5 includes juvenile myelomonocytic leukemia (JMML) within the category of MPN, while the ICC includes JMML in a separate group of pediatric myeloid neoplasms (discussed later).

Like the WHO-HAEM4R, the ICC recognizes an accelerated phase of CML (CML-AP), but this has been simplified from WHO-HAEM4R CML-AP definition to now only include cases with 10–19% blasts, ≥20% blood basophils, and/or presence of certain specific clonal cytogenetic aberrations in addition to the defining *BCR::ABL1* rearrangement. In contrast, the WHO-HAEM5 does not recognize CML-AP, but instead defines high-risk morphologic and genetic features within chronic phase CML. In both classifications, blast phase CML is still defined by ≥20% blasts. There are essentially no differences in the diagnostic criteria for the *JAK2/MPL/CLAR*-associated MPN and chronic eosinophilic leukemia between the two classifications, and both retain a category to place MPN that cannot be otherwise classified, but with slightly different names: MPN-NOS in WHO-HAEM5 and MPN-unclassifiable in ICC. CNL is strongly associated with a somatic *CSF3R* mutation and in recognition of this strong genotype-phenotype association, the ICC allows a diagnosis of CNL in the presence of *CSF3R* mutation with a WBC ≥13 × 10^9^/L provided other criteria are met, while the WHO-HAEM5 continues to require a WBC ≥25 × 10^9^/L for all cases, as in WHO-HAEM4R. This difference is expected to affect very few cases given the rarity of CNL and its strong association with a markedly elevated WBC [17, 18] ; it may allow an earlier diagnosis for the prevalent *CSF3R*-mutated cases when following the ICC criteria.

### MDS

In addition to a different name for the overall disease group, WHO-HAEM5 and ICC have several differences in the criteria that define the borders of MDS as well as the division of MDS into distinct subtypes.

#### Borders of MDS with myeloid neoplasm precursor lesions

In the WHO-HAEM5, morphologic dysplasia affecting at least 10% of cells in at least one hematopoietic lineage is required to establish a diagnosis of MDS in all instances; in the ICC, similar to WHO-HAEM4R, there are several genetic aberrations that are considered to define MDS in a patient with unexplained cytopenia, even in the absence of ≥10% dysplasia. These aberrations are now limited to the presence of complex karyotype (at least 3 independent acquired cytogenetic abnormalities, excluding -Y), -7/del(7q), del(5q), and *SF3B1* or bi-allelic *TP53* mutations. The latter two mutations must be seen at a minimum VAF of at least 10%, since small CH clones would be unlikely to cause a clinically significant cytopenia. Importantly, the above genetic abnormalities are almost ubiquitously associated with significant morphologic dysplasia and thus it is expected that this difference will result in few discrepancies. In practice, the absence of dysplasia in the setting of these MDS-associated abnormalities is more likely to reflect a suboptimal sample rather than truly absent morphologic dysplasia [19].

#### Borders of MDS with AML

Both WHO-HAEM5 and ICC recognize several genetic lesions as AML-defining (see AML section below). However, the ICC requires at least 10% blasts in bone marrow or blood to classify any case as AML, whereas WHO-HAEM5 allows any increase in blasts to qualify for AML in the presence of an AML-defining genetic lesion; although increased blasts is typically defined as ≥5% in bone marrow or ≥2% in blood, there is no clear evidence to support a specific blast cutoff in this context. Given some subjectivity in counting blasts, cases which yield discrepant diagnoses due to these different blast thresholds should be approached with careful clinical correlation and follow-up, with the treatment approach influenced by the clinical picture as well as the specific blast count at a given timepoint [20]. Conversely, while WHO-HAEM5 requires at least 20% blasts to define AML in the absence of an AML-defining genetic lesion, the ICC recognizes an “MDS/AML” overlap group encompassing cases with 10–19% blasts that lack AML-defining genetics, effectively replacing MDS-EB2. The rationale behind this change in the ICC is that some patients with MDS/AML may benefit from AML-type intensive therapy, and this designation may facilitate wider therapeutic options for patients with 10–19% blasts [21]. The ICC recommends to subclassify MDS/AML along the lines of other AML, into 4 subgroups defined by mutated *TP53*, myelodysplasia-related gene mutations, myelodysplasia-related cytogenetic abnormalities, or no specific genetic features (NOS); further research is needed to determine the clinical significance of subgrouping MDS/AML and the relationship of these subgroups to their overt AML counterparts with ≥20% blasts [22]. All recurrent AML-defining genetic aberrations are classified as overt AML and are therefore excluded from MDS/AML.

#### MDS classification

Both WHO-HAEM5 and ICC have recognized *SF3B1* mutation and bi-allelic *TP53* mutation as defining new MDS subtypes, while retaining isolated del(5q) as a specific MDS subtype. However, there are several minor differences in the definitions of the new *SF3B1* and *TP53* entities, which are shown in Table 3. Cases with excess (≥5% in bone marrow and/or ≥2% in blood) blasts are categorized using different terminology from the prior WHO-HAEM4R: MDS with excess blasts and MDS/AML in ICC, and MDS with increased blasts-1 and MDS with increased blasts-2 in WHO-HAEM5, correspond respectively to the prior MDS with excess blasts-1 and MDS with excess blasts-2. However, there are some minor differences in these correspondences, as shown in Table 3. Given that fibrosis has been shown to confer adverse prognosis in MDS [23], the WHO-HAEM5 (but not the ICC) introduced a new subgroup of MDS with increased blasts: “MDS with increased blasts and fibrosis”. For cases that lack excess blasts or Auer rods and do not qualify for any of the three genetically-defined groups [*SF3B1*, bi-allelic *TP53*, or del(5q)], the ICC subdivides cases by the presence of dysplasia involving one (single lineage dysplasia, SLD) or more (multilineage dysplasia, MLD) hematopoietic lineages, while the WHO-HAEM5 introduced a new entity of hypoplastic MDS (MDS-h), defined by age-adjusted hypocellularity (cellularity < 20% for patients ≥70 years and < 30% for patients < 70 years). Although genetically heterogeneous, MDS-h cases may have a more favorable prognosis and respond more effectively to immunosuppressive therapy compared to other MDS lacking increased blasts [24]. The WHO-HAEM5 has also retained ring sideroblasts in the absence of *SF3B1* mutation as a morphologically-defined entity, although recent studies have shown similar prognosis to cases of MDS with low blasts that lack ring sideroblasts [25]. WHO-HAEM5 removed requirement for SLD vs. MLD distinction due to poor reproducibility of this subjective determination [16], while the ICC retained it due to prognostic relevance in multiple studies [26, 27].

**Table 3:** Comparison of WHO-HAEM5 and ICC classification of adult MDS

Genetic/morphologic feature	WHO-HAEM5	ICC	Differences between WHO-HAEM5 and ICC
*SF3B1* mutation	MDS with low blasts and *SF3B1* mutation	MDS with mutated *SF3B1*	• ICC requires *SF3B1* VAF of ≥10%, WHO requires VAF of ≥5% • ICC excludes cases with abnormal 3q26.2 and *RUNX1* mutation
*TP53* mutation	MDS with biallelic *TP53* inactivation	MDS with mutated *TP53*	• ICC requires *TP53* VAF of ≥10%, WHO has no minimal VAF • ICC allows mono-allelic *TP53* mutation for cases with 10–19% blasts (MDS/AML), WHO requires bi-allelic mutation for all cases • ICC, but not WHO allows complex karyotype to qualify for bi-allelic mutation if *TP53* LOH status is unknown
Del(5q)	MDS with isolated deletion (5q)	MDS with del(5q)	• WHO, not ICC, requires dysplasia in at least 10% of cells in at least 1 lineage
Blast excess or Auer rods	MDS with increased blasts-1 (MDS-IB1) MDS with increased blasts-2 (MDS-IB2) MDS with increased blasts and fibrosis (MDS-F)	MDS with excess blasts (MDS-EB) MDS/AML	WHO IB2 mostly equivalent to MDS/AML and WHO IB1 mostly equivalent to MDS-EB. However: • Cases with Auer rods and < 10% blasts are MDS-EB in ICC and MDS-IB2 in WHO • Cases with 5–9% PB blasts are MDS-EB in ICC and MDS-IB2 in WHO • WHO MDS-F corresponds to ICC MDS-EB and MDS/AML cases with grade 2–3 fibrosis
No blast excess	MDS with low blasts MDS, hypoplastic MDS with low blasts and ring sideroblasts	MDS-NOS-SLD MDS-NOS-MLD	WHO subdivides these cases based on marrow hypocellularity or ≥15% ring sideroblasts; ICC subdivides these cases based on dysplasia in 1 versus 2–3 hematopoietic lineages.

*Abbreviations* LOH, loss of heterozygosity; SLD, single lineage dysplasia; MLD, multi-lineage dysplasia

### Myeloid neoplasms in Children

In both WHO-HAEM5 and ICC, the above MDS classifications apply to adult patients (age ≥18 years), and both classify pediatric MDS separately. Although both classifications employ different names for specific entities, these entities are mostly analogous to one another and have similar diagnostic criteria (Table 4). Of note, the ICC MDS/AML entity does not apply to pediatric MDS: pediatric MDS patients with increased blasts are managed differently from adult MDS patients, and may not warrant intensive therapy prior to stem cell transplant despite elevated blast counts approaching AML.

**Table 4:** Comparison of WHO-HAEM5 and ICC classification of MDS and JMML in children

WHO-HAEM5	ICC	Differences between WHO-HAEM5 and ICC
Childhood MDS with low blasts, hypocellular	Refractory cytopenia of childhood	• WHO-HAEM5 allows ≥10% dysplasia in any lineage, while ICC requires ≥10% dysplasia specifically in megakaryocytes (or lesser degrees of dysplasia in 2 or 3 lineages)
Childhood MDS with low blasts	MDS-NOS	• WHO-HAEM5 requires cytopenia and ≥10% dysplasia, while ICC allows absence of cytopenia or dysplasia if an MDS-defining cytogenetic abnormality is present.
Childhood MDS with increased blasts	MDS with excess blasts	• None
Juvenile myelomonocytic leukemia (JMML)	Juvenile myelomonocytic leukemia (JMML)	• WHO-HAEM5 allows cases lacking RAS-pathway mutations in the presence of increased HbF, leukoerythroblastosis, thrombocytopenia with hypercellular marrow, or hypersensitivity of myeloid progenitors to GM-CSF, while ICC excludes such cases and instead classifies them as JMML-like neoplasms.

Regarding juvenile myelomonocytic leukemia (JMML), both classifications removed this entity from the prior MDS/MPN group. The ICC now considers JMML in a group of pediatric myeloid neoplasms including pediatric MDS, while the WHO-HAEM5 has placed JMML in the MPN group. Both WHO-HAEM5 and ICC have similar definitions for JMML, except the ICC considers the presence of RAS-pathway mutations an absolute requirement for the diagnosis; related cases that lack a RAS-pathway mutation are considered within a separate entity of JMML-like neoplasms.

### MDS/MPN

#### Chronic myelomonocytic leukemia (CMML)

Major changes were introduced to CMML diagnostic criteria in both WHO-HAEM5 and ICC, mainly lowering the threshold of absolute monocytosis to 0.5 × 10^9^/L in PB, while still requiring that monocytes comprise at least 10% of WBCs. This was based on recent evidence showing that patients with relative monocytosis (≥10% of WBCs) but absolute monocytosis in the 0.5-<1 × 10^9^/L range (so-called ‘oligomonocytic CMML’) displayed similar features to ‘traditional’ CMML with monocytes ≥1 × 10^9^/L [28, 29]. Additionally, the subgroup of CMML-0 (< 2% blasts in blood and < 5% blasts in bone marrow) introduced in the WHO-HAEM4R, that was previously thought to have relatively indolent behavior [30], has been eliminated due to its limited prognostic impact and poor reproducibility based on additional more comprehensive data [31]. Both WHO-HAEM5 and ICC require evidence of clonality for the diagnosis of oligomonocytic CMML and both continue to subdivide all CMML into myelodysplastic and myeloproliferative subtypes based on a WBC threshold of 13 × 10^9^/L. However, there are several differences between WHO-HAEM5 and ICC CMML criteria (Table 5).

**Table 5:** Chronic myelomonocytic leukemia diagnostic criteria

Criteria for diagnosis	WHO-HAEM5	ICC
Cytosis	Monocytes ≥0.5 × 10^9^/L and ≥10% of the WBC	
Cytopenia	Not required	At least one cytopenia
Blasts	CMML-1: <10% BM and < 5% PB CMML-2: 10–19% BM or 5–19% PB	
Morphology	No specific BM morphology required	BM hypercellularity due to a myeloid proliferation, often with increased monocytes
Cases with monocytes ≥1 × 10^9^/L	One of the following: 1. Dysplasia 2. Abnormal monocyte partitioning 3. Clonal genetic abnormality	One of the following: 1. Dysplasia 2. Increased blasts 3. Abnormal monocyte immunophenotype 4. Clonal genetic abnormality (VAF≥10%)
Cases with monocytes 0.5-<1 × 10^9^/L	Both of the following: 1. Dysplasia 2. Clonal genetic abnormality and dysplasia	Clonal genetic abnormality (VAF≥10%)
1. Exclusion	CML, other MPN and M/LN-TK fusions	
2. Subtyping	Myelodysplastic (WBC < 13 × 10^9^/L) and myeloproliferative (WBC≥13 × 10^9^/L)	

*Abbreviations* M/LN-TK, Myeloid/lymphoid neoplasms with eosinophilia and TK fusion

1. The ICC emphasizes the presence of at least one cytopenia as a prerequisite for diagnosing CMML, while noting that a small proportion of cases may show only borderline or no cytopenia, usually in early-phase disease.
2. Characteristic bone marrow morphology (hypercellular marrow with myeloid predominance, often with increased monocytes) is required by ICC but not WHO-HAEM5. Consequently, some patients who meet WHO-HAEM5 CMML diagnostic criteria but do not show typical bone marrow morphologic features could be classified as clonal monocytosis of undetermined significance (CMUS) or clonal cytopenia and monocytosis of undetermined significance (CCMUS), or potentially as MDS if there is sufficient dysplasia [32]. Future studies are needed to address these discrepancies, particularly in relation to the typical CMML mutation profile of *ASXL1*,* SRSF2*, and *TET2* mutations (often including bi-allelic *TET2* lesions).
3. Although both WHO-HAEM5 and ICC employ monocyte immunophenotype in supporting a diagnosis of ‘classic’ CMML, the ICC allows any immunophenotypic aberrancy (such as expression of CD56 and/or CD2), while the WHO-HAEM5 specifies abnormal monocyte partitioning defined by CD16 and CD14 [33, 34].
4. The ICC but not WHO-HAEM5, requires ≥10% VAF for mutations supporting a diagnosis of CMML.
5. Lastly, *NPM1* mutation is considered as AML defining by WHO-HAEM5 in cases with increased blasts/blasts equivalent but otherwise meeting criteria for CMML, while ICC still retains *NPM1*-mutated CMML for cases with < 10% blasts or cases with a prior history of CMML that secondarily acquire an *NPM1* mutation.

A recent study suggests that clonal monocytosis, CMML, and MDS exist on a spectrum, and the complex diagnostic criteria put forth by both WHO-HAEM5 and ICC may arbitrarily separate biologically related entities [32]. Thus, further research is needed to optimize the classification of clonal proliferations associated with cytopenia and variable monocytosis and these criteria may evolve in future myeloid neoplasm classifications.

### MDS/MPN with iso17q is a new provisional entity in ICC

In the ICC, MDS/MPN with i(17q) is added as a new provisional subentity under the diagnostic umbrella of MDS/MPN-NOS. This category includes cases meeting criteria for MDS/MPN-NOS (i.e. failing to fulfill criteria for MDS or other MDS/MPN entities), but with an i(17q) cytogenetic abnormality with up to one additional cytogenetic abnormality (non-complex karyotype) other than del(7q)/−7. These cases show a high frequency of mutations in *SRSF2*, *SETBP1*, *ASXL1*, and *NRAS* genes [35]. *SRSF2* is often co-mutated with *SETBP1* (but not with *TET2*) and co-existent triple mutations in *SRSF2*, *SETBP1*, and *ASXL1* are seen in approximately 30% of cases. Despite loss of one *TP53* locus on 17p due to the i(17q), *TP53* mutations are absent in this entity.

### Other changes

Although the criteria remain nearly identical, WHO-HAEM5 renamed “atypical chronic myeloid leukemia” to “MDS/MPN with neutrophilia” with the intention of avoiding potential confusion with CML. The WHO-HAEM4R entity “MDS/MPN with ring sideroblasts and thrombocytosis” (MDS/MPN-RT-T) has been largely redefined based on the highly prevalent *SF3B1* mutation in these cases, and is renamed “MDS/MPN with *SF3B1* mutation and thrombocytosis” in both WHO-HAEM5 and ICC. However, “MDS/MPN with ring sideroblasts and thrombocytosis” has been retained as a repository for cases with wild-type *SF3B1* and ≥15% ring sideroblasts in both ICC and WHO-HAEM5, as the clinical behavior and biologic features of these infrequent cases is uncertain.

### AML

There are major updates on the classification of AML in both WHO-HAEM5 and ICC.

#### Diagnostic algorithm

Both WHO-HAEM5 and ICC classifications emphasize the importance of genetic findings and their influence on the disease biology. The category of AML with recurrent genetic abnormalities is expanded by including more recurrent cytogenetic rearrangements that lead to novel fusion genes and/or increased oncogene expression driving leukemogenesis (Table 6). The terminology of AML with myelodysplasia related changes (AML-MRC) is replaced by AML, myelodysplasia-related (AML-MR) in WHO-HAEM5, representing a single entity defined by the presence of at least one of the following: history of MDS or MDS/MPN, MR cytogenetic abnormalities and/or MR gene mutations (Table 7). This AML-MR group corresponds to 3 separate AML entities in the ICC: those defined by MR gene mutations (with or without MR cytogenetics abnormalities), MR cytogenetic abnormalities (without MR gene mutations), or mutated *TP53* (mono- or bi-allelic, and with VAF ≥10%, since the vast majority of TP53-mutated AML cases have complex karyotype that qualifies for AML-MR per WHO-HAEM5). Additionally, there are some differences in the composition of MR gene mutations and MR cytogenetic abnormalities between WHO-HAEM5 and ICC (Table 7). The ICC removed history of MDS or MDS/MPN as classifier for AML, and applies this history as a disease qualifier to the genetically-defined AML subtype; since most cases of AML progressed from MDS or MDS/MPN will have MR mutations and/or cytogenetic abnormalities, or fall into the *TP53*-mutated AML category in the ICC, these cases will still largely be in concordance with the AML-MR WHO-HAEM5 category. Due to its poor interobserver reproducibility and often difficult applicability [36], morphologic dysplasia was removed as a diagnostic criterion for AML-MR in both WHO-HAEM5 and ICC.

**Table 6:** Updates on blast cutoff in AML

	WHO-HAEM4R	WHO-HAEM5	ICC
AML with recurrent genetic abnormalities*			
• Acute promyelocytic leukemia (APL) with t(15;17)(q24.1;q21.2)/ PML::RARA** • AML with t(8;21)(q22;q22.1) /*RUNX1::RUNX1T1* • AML with inv(16)(p13.1q22) or t(16;16)(p13.1;q22)/*CBFB::MYH11*	No blast cutoff	Increased blasts	Blasts ≥ 10%
• AML with t(x;11)(x; q23.3)/ *KMT2A* rearrangements*** • AML with t(6;9)(p22.3;q34.1)/*DEK::NUP214* • AML with inv(3)(q21.3q26.2) or t(3;3)(q21.3;q26.2)/*GATA2;MECOM(EVI1)* **** • AML with other *MECOM* rearrangements • AML with *NUP98* rearrangements • AML with *RBM15::MRTFA* fusion*****	Blasts ≥20%	Increased blasts	Blasts≥10%
• AML with other rare recurring translocations	Blasts≥20%	Blasts≥20%	Blasts≥10%
• AML with *BCR::ABL1* fusion	Blasts≥20%	Blasts≥20%	Blasts≥20%
• AML with *NPM1* mutation	Blasts≥20%	Increased blasts	Blasts≥10%
• AML with *CEBPA* mutation	Blasts≥20% (bi-allelic)	Blasts≥20% (bi-allelic or bZIP)	Blasts≥10% (in frame bZIP only)
AML with mutated *TP53*	Not included	Not included	Blasts≥20% VAF≥10%
AML-MR	Blasts≥20% (AML-MRC)	Blasts≥20%	Not included
• AML with MR gene mutations	Not included	Not included	Blasts≥20%
• AML with MR cytogenetic abnormalities	Not included	Not included	Blasts≥20%
AML-NOS / AML defined by differentiation	Blasts≥20%	Blasts≥20%	Blasts≥20%
MDS with 10–19% blasts	MDS-EB2	MDS-IB2	MDS/AML

*WHO-HAEM5 uses a shorter nomenclature without listing cytogenetic changes but adds “fusion” or “rearrangement” to the nomenclature when appropriate

**ICC lists “AML with other *RARA* rearrangements” separately

***ICC separates “AML with t(9;11)(p21.3;q23.3)/*MLLT3::KMT2A*” from “AML with other *KMT2A* rearrangements”

****WHO-HAEM5 combines “AML with inv(3)(q21.3q26.2) or t(3;3)(q21.3;q26.2)/*GATA2;MECOM(EVI1)*” and “AML with other *MECOM* rearrangements” into “AML with *MECOM* rearrangement”

***** This is listed under “AML with other rare recurring translocations” by ICC

**Table 7:** MR genes and MR cytogenetic abnormalities

	Genetics	Differences between WHO-HAEM5 and ICC
MR gene mutations	• *SRSF2*,* SF3B1*,* U2AF1*,* ZRSR2*,* ASXL1*,* EZH2*,* BCOR*,* STAG2*, ***RUNX1***	• ICC includes *RUNX1*, while WHO-HAEM5 does not.
MR cytogenetic abnormalities	• Complex karyotype (≥ 3 abnormalities) • del(5q)/t(5q)/add(5q) • -7/del(7q) • del(12p)/t(12p)/add(12p) • -17/del(17p)/add(17p) • i(17q) • idic(X)(q13) • **del(11q)** • **Monosomy 13 or del(13q)** • **+ 8** • **Del(20q)**	• WHO-HAEM5, not ICC, includes del(11q) and − 13 or del(13q). • ICC, not WHO-HAEM5, includes + 8 and del(20q) • ICC complex karyotype excludes hyperdiploid karyotypes with three or more trisomies (or polysomies) without structural abnormalities.

AML cases that fail to place in any of the aforementioned genetic categories are classified as “AML defined by differentiation” in the WHO-HAEM5, further refined by their specific immunophenotypic profile (myeloid, monocytic, megakaryocytic, or erythroid), and as “AML-NOS” in the ICC. One subcategory of WHO-HAEM5 AML defined by differentiation, acute erythroid leukemia (AEL, previously termed ‘pure erythroid leukemia in WHO-HAEM4R), nearly ubiquitously harbors bi-allelic *TP53* mutations and complex karyotype and thus corresponds to AML with mutated TP53 in the ICC. Since AEL supersedes AML-MR in WHO-HAEM5, these rare cases are divergently classified in WHO-HAEM4R and ICC.

**Fig. 2:**
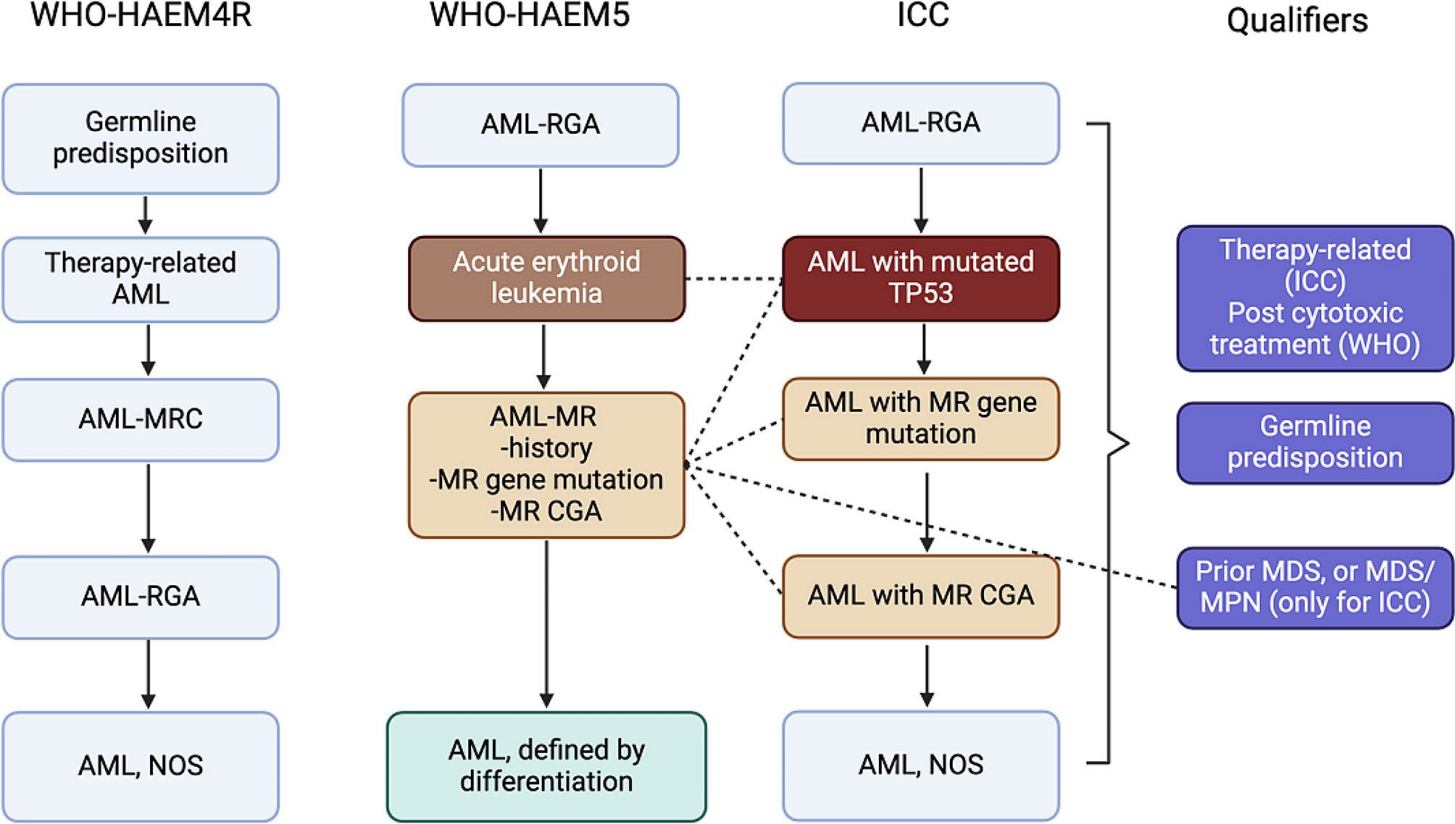
Algorithmic updates of AML classification. AML-RGA, AML with recurrent genetic abnormalities. AML-MRC, AML with myelodysplasia-related changes. AML-MR, AML, myelodysplasia-related. MR CGA, myelodysplasia related cytogenetic abnormalities. NOS, not otherwise specified

Both WHO-HAEM5 and ICC now apply therapy-relatedness as a qualifier to the genetic/differentiation AML subtype, except the WHO-HAEM5 has changed “therapy-related” terminology to “post-cytotoxic treatment”, since a prior history of cytotoxic therapy does not necessarily imply a causation. Both WHO-HAEM5 and ICC also consider germline predisposition as disease qualifiers to the relevant AML subtype, e.g. AML with MR gene mutation, in the setting of germline RUNX1 mutation. A detailed comparison of WHO-HAEM5 and ICC AML diagnostic algorithms is shown in Fig. 2.

#### Blast cutoff

The blast cutoff for AML diagnosis has been continually evolving. In the original FAB Classification, patients with myelodysplastic syndromes and 20–29% blasts were classified as refractory anemia with excess blasts in transformation (RAEB-T). In 2001, WHO-HAEM3 adopted a blast cutoff of 20% for AML diagnosis, thus eliminating RAEB-T and encompassing them within AML. This cutoff has since remained largely unchanged with an exception of AML with *PML::RARA* and AML with the core-binding factor gene translocations inv(16)/t(16;16) or t(8;21), in which the presence of such rearrangements are considered as pathognomonic for AML regardless of the blast percentage. As discussed above, both the WHO-HAEM5 and ICC have softened the blast requirement for most genetic subtypes of AML (Table 6), with the exception of *BCR::ABL1* fusion: cases with *BCR::ABL1* and 10–19% blasts are still considered within the category of CML (accelerated phase in the ICC).

#### Other changes

##### AML with CEBPA mutations

Both WHO-HAEM5 and ICC further refined the diagnostic criteria for AML with *CEBPA* mutations based on recent studies showing that the favorable prognostic impact is determined by the presence of an in-frame bZIP mutation in the gene, not merely the presence of two (bi-allelic) mutations [37, 38]. The ICC requires the presence of at least one in-frame bZIP mutation for diagnosing this entity, while in WHO-HAEM5, AML with CEBPA mutation is defined more broadly by either any single bZIP mutation or any biallelic mutations. Additionally, while the ICC allows a diagnosis of AML with CEBPA mutation with ≥10% blasts (similar to other genetically-defined AML, discussed above), the WHO-HAEM5 requires 20%, since the rare cases of bZIP CEBPA-mutated disease presenting with < 20% blasts have not been well studied.

### Myeloid/lymphoid neoplasms with tyrosine kinase gene fusions

The category name is changed from the prior “myeloid and lymphoid neoplasms with eosinophilia (M/LN-eo) and gene rearrangement” to “Myeloid/lymphoid neoplasms with eosinophilia and tyrosine kinase gene fusions” (M/LN-eo-TK) by both WHO-HAEM5 and ICC (Table 8). M/LN-eo-TK often manifests as chronic myeloid neoplasms but can present as AML, B-ALL, T-ALL or even MPAL. Inclusion of this group of diseases in the differential diagnosis of chronic myeloid neoplasms and acute leukemias and detection of the defining TK fusions are key for an accurate and timely diagnosis, since many of these entities are effectively treated by targeted therapies. In addition to previously included *PDGFRA*,* PDGFRB*,* FGFR1*, and *JAK2* fusions, *FLT3* fusions and *ETV6::ABL1* are now added to this category in both WHO-HAEM5 and ICC [39–41]. The most common partner gene of *FLT3* fusions is *ETV6* located at 12p13 [42]. *PDGFRA*,* PDGFRB* and *ETV6::ABL1* cases are sensitive to ABL1 inhibitors. WHO-HAEM5 also created a subgroup named MLN-eo with other defined tyrosine kinase fusions to encompass other rare tyrosine kinase fusions i.e. *ETV6::FGFR2*; *ETV6::LYN*; *ETV6::NTRK3*; *RANBP2::ALK*; *BCR::RET*; and *FGFR1OP::RET*.

**Table 8:** Myeloid/lymphoid neoplasms with eosinophilia and TK fusion

WHO-HAEM5	ICC	Most common fusion	Typical clinical and BM manifestations	Targeted therapy
*PDGFRA*	*PDGFRA*	Cryptic deletion at 4q12/ *FIP1L1::PDGFRA*	Common: CEL-like BM with frequent extramedullary involvement Others: B-ALL/LL, AML or mast cell proliferations	Excellent response to TKI
*PDGFRB*	*PDGFRB*	t(5;12)(q32;p13.2)/ *ETV6::PDGFRB*	Common: CEL-like or monocytosis with eosinophilia Others: ALL, AML or mast cell proliferations	Excellent response to TKI
*FGFR1*	*FGFR1*	t(8;13)(p11.2;q12.1)/ *ZMYM2::FGFR1*	Common: Extramedullary T-ALL/LL with BM MPN-like or blast phase of MPN; Trilineage MPAL not uncommon	High rate of response to FGFR inhibitor such as pemigatinib, especially for cases in chronic phase
*JAK2*	*JAK2*	t(8;9)(p22;p24.1)/ *PCM1::JAK2*	Often show characteristic pronormoblast clusters	Limited responses to ruxolitinib
*FLT3*	*FLT3*	t(12;13)(p13.2;q12.2)/ *ETV6::FLT3*	T-ALL/LL or myeloid sarcoma with CEL-like or MDS/MPN BM features	Various responses to specific FLT3 inhibitors
*ETV6::ABL1*	*ETV6::ABL1*	t(9;12)(q34.1;p13.2)/ *ETV6::ABL1*	CML-like with frequent eosinophilia in chronic or blast phase	Good response to TKI in chronic phase
Other defined tyrosine kinase fusions	Not included	*ETV6::FGFR2; ETV6::LYN; ETV6::NTRK3; RANBP2::ALK; BCR::RET; FGFR1OP::RET*	Variable	Unknown

*Abbreviations* CEL, chronic eosinophilic leukemia; TKI, tyrosine kinase inhibitor; ALL, acute lymphoblastic leukemia; MPAL, mixed phenotype acute leukemia

### Systemic mastocytosis

WHO-HAEM5 and ICC both made only minimal refinements to the definition of systemic mastocytosis (SM). While the WHO-HAEM5 allows any hematologic neoplasm (including lymphoma and plasma cell myeloma) within the entity of “SM with an associated hematologic neoplasm” (SM-AHN), the ICC specifically restricts this category to myeloid neoplasms and renames the entity “SM with associated myeloid neoplasm” (SM-AMN); this was based on demonstrated shared genetic origin between co-occurrent myeloid, but not lymphoid neoplasms, with the mast cell clone [43]. Another difference is that the ICC requires immature mast cell cytomorphology for mast cell leukemia (MCL), while the WHO-HAEM5 MCL category encompasses rare cases displaying well-differentiated morphology, terming them “chronic MCL” as retained from the prior WHO-HAEM4R [44].

### Hematologic/myeloid neoplasms with germline predisposition

Comparing to WHO-HAEM4R, there are subtle changes in WHO-HAEM5 and ICC and minor differences in nomenclature for the category of germline predisposition disorders, which was first introduced into the WHO-HAEM4R classification (Table 9). Several additional genes are incorporated into this group (Table 10): germline *TP53* mutations, RASopathies, germline *SAMD9/SAMD9L* mutations, and germline *BLM* mutations. In ICC the title is changed from “myeloid neoplasms” to “hematologic neoplasms” with germline predisposition as increasing data have demonstrated that many of these germline-mutated genes predispose not only to myeloid malignancy but also to lymphoid malignancies [45]. In addition to the genes mentioned above, the ICC added a new subgroup: acute lymphoblastic leukemia with germline predisposition encompassing patients with germline *PAX5* and *IKZF1* mutations.

**Table 9:** Hematologic/myeloid neoplasms with germline predisposition

WHO-HAEM5	ICC	Entities included in both WHO-HAEM5 and ICC	Differences between WHO-HAEM5 and ICC
Myeloid neoplasms with germline predisposition without a preexisting platelet disorder or organ dysfunction	Hematologic neoplasms with germline predisposition without a constitutional disorder affecting multiple organ systems	Germline *CEBPA*, *DDX41* and *TP53* mutations	None
Myeloid neoplasms with germline predisposition and pre-existing platelet disorder	Hematologic neoplasms with germline predisposition associated with a constitutional platelet disorder	Germline *RUNX1*, *ANKRD26* and *ETV6* mutations	None
Myeloid neoplasms with germline predisposition and potential organ dysfunction	Hematologic neoplasms with germline predisposition associated with a constitutional disorder affecting multiple organ systems	Germline *GATA2* Germline *SAMD9/SAMD9L* Bone marrow failure syndromes Down syndrome RASopathies (JMML with *NF1*,* CBL*)	WHO (not ICC): Germline *BLM* mutations ICC (not WHO): Diamond-Blackfan anemia
Not included	Acute lymphoblastic leukemia with germline predisposition		ICC group includes germline *PAX5* and *IKZF1* mutations.

**Table 10:** ALAL/MPAL

WHO-HAEM5	ICC
ALAL with defining genetic abnormalities	MPAL with defining genetic alterations
MPAL with *BCR::ABL1* fusion	MPAL with *BCR::ABL1*
MPAL with *KMT2A* rearrangement	MPAL with t(v;11q23.3); *KMT2A* rearranged
ALAL with other defined genetic alterations	
MPAL with *ZNF384* rearrangement	MPAL with *ZNF384* rearrangement
ALAL with *BCL11B* rearrangement	MPAL with *BCL11B* activation
ALAL, immunophenotypically defined	MPAL with defining immunophenotypic changes
MPAL, B/myeloid	B/myeloid MPAL
MPAL, T/myeloid	T/myeloid MPAL
MPAL, rare types	B/T/myeloid MPAL and B/T MPAL
ALAL, NOS	ALAL-NOS
Acute undifferentiated leukemia (AUL)	Acute undifferentiated leukemia (AUL)

### Acute leukemias of ambiguous lineage (ALAL)/mixed phenotype acute leukemias (MPAL)

The classification updates on ALAL/MPAL are highly concordant between WHO-HAME5 and ICC. ALAL/MPAL is divided into two groups: ALAL/MPAL with defining genetic abnormalities and ALAL/MPAL-NOS or immunophenotypically defined [7, 46, 47] (Table 10). The former includes cases with *BCR::ABL1* and *KMT2A* rearrangements (both also previously recognized by WHO-HAEM4R) and two new entities: MPAL with *ZNF384* rearrangement and ALAL/MPAL with *BCL11* rearrangement/activation.

*ZNF384*-rearranged MPAL compromises nearly half of MPAL with B/myeloid immunophenotype, and approximately 20% of all MPAL cases [48], and is particularly common in children. Partners include *TCF3*,* EP300*,* TAF15* and *CREBBP*^48^. *ZNF384*-rearranged B/myeloid MPAL is transcriptionally similar to its B-ALL counterpart, suggesting a biological continuum in this disease. *BCL11B*-rearranged ALAL compromises one third of MPAL with T/myeloid immunophenotype, and 10–15% of all MPAL; rare cases present as acute undifferentiated leukemia. FISH studies show translocations involving the *BCL11B* gene at 14q32, with partners including 2q22 (*ZEB2*), 6q25 (*ARID1B*), 7q21(*CDK6*) and 8q24 (*BENC-MYC*). *BCL11B* rearrangements are also observed in a subset of ETP-ALL and rarely AML-NOS/AML minimally differentiated (M0/M1) cases, also suggesting immunophenotypic variability within this genetic biologic entity [49, 50]. *BCL11B*-rearranged ALAL may be sensitive to FLT3 and JAK-STAT inhibitors [50] although this approach has not yet been studied clinically.

#### Boundary between AML-MR and ALAL/MPAL

According to WHO-HAEM4R a diagnosis of AML-MRC or therapy-related AML overrode a diagnosis of ALAL/MPAL, even when a mixed immunophenotype was present [47]. However, changes in the diagnostic criteria for AML by WHO-HAEM5 and ICC create new dilemmas [6, 7]. Specifically, the criteria for AML-MR have been modified in both WHO-HAEM5 and ICC to include MR gene mutations, regardless of history of antecedent hematologic malignancy or myelodysplasia-related cytogenetic abnormalities, which would potentially shift more cases previously classified as MPAL to AML-MR. Therefore, it is uncertain how these changes will shift the boundary between AML-MR/t-AML and MPAL, which requires clarification in future studies [50]. The ICC stipulates a minimum of 5% population of divergent aberrant lineage to establish a diagnosis of MPAL, while the WHO-HAEM5 classification does not stipulate a specific minimal threshold.

### Blastic plasmacytoid dendritic cell neoplasm (BPDCN)

In WHO-HAEM5, two entities composed of plasmacytoid dendritic cells are recognized: mature plasmacytoid dendritic cell proliferation (MPDCP) and blastic plasmacytoid dendritic cell neoplasm.

MPDCP are clonal proliferations of plasmacytoid dendritic cells (PDCs) that occur in association with myeloid neoplasms, most often CMML, and involve the skin, bone marrow or lymph nodes with mature bland cytologic features [53–55]. MPDCP has also been recently described in AML, particularly with *RUNX1* mutations [55, 56]. In this setting, the morphology of PDCs ranges from mature to immature and at the extreme may be indistinguishable from BPDCN involving marrow. The ICC does not formally recognize MPDCP as a distinct myeloid neoplasm, given its typical association with other myeloid neoplasms. BPDCN is retained in both ICC and WHO-HAEM5, with essentially identical definition to BPDCN in WHO-HAEM4R.

### B lymphoblastic leukemia/lymphoma (B-ALL/LBL)

Although most B-ALL/LBL subtypes from the WHO-HAEM4R are retained, both WHO-HAEM5 and ICC include new entities subsequently identified by gene expression profiling and clustering algorithms (Table 11). These new entities are characterized by distinct clinical behavior/features and are driven by gene rearrangements, point mutations or gene expression signatures.

**Table 11:** B lymphoblastic leukemia/lymphoma (B-ALL/LBL)

B-ALL entities	Differences between WHO-HAEM5 and ICC
NOS	Same
with hyperdiploidy	Same
with hypodiploidy	ICC separates this into two subentities: • low hypodiploid • near haploid
with *iAMP21*	Same
with t(9;22)(q34;q11.2); *BCR::ABL1*	ICC separates this into two subentities: • with lymphoid only involvement • with multilineage involvement
*BCR::ABL1*-like	ICC separates this into three subentities: • *ABL1* class rearranged • JAK-STAT activated • NOS
with t(12;21)(p13.2;q22.1); *ETV6::RUNX1*	Same
*ETV6::RUNX1-*like*#	Same
with t(1;19)(q23;p13.3); *TCF3::PBX1*	Same
with t(v;11q23.3); *KMT2A* rearranged	Same
with t(5;14)(q31.1;q32.1); *IGH :: IL3*	Same
with *HLF* rearrangement*	WHO-HAEM5 only lists *TCF3::HLF* fusion ICC includes other *HLF* rearrangements
with *DUX4* rearrangement*	Same
with *MEF2D* rearrangement*	Same
with *MYC* rearrangement*	Same
with *NUTM1* rearrangement*	Same
with PAX5 p.P80R*	Same
with *PAX5*alt*	Same
with *ZNF384* rearrangement*	Same
with *UBTF::ATXN7L3/PAN3*,* CDX2* (“*CDX2*::*UBTF*”)*	Only included in ICC, but not WHO-HAEM5
with mutated IKZF1 N159Y*	Only included in ICC, but not WHO-HAEM5
with mutated ZEB2 (p.H1038R)/IGH::CEBPE*	Only included in ICC, but not WHO-HAEM5
*ZNF384* rearranged-like*#	Only included in ICC, but not WHO-HAEM5
*KMT2A* rearranged-like*#	Only included in ICC, but not WHO-HAEM5

*New entities, not included in WHO-HAEM4R

#Recognized by gene expression profiles

#### Changes to previously recognized entities

The previously recognized B-ALL/LBL entities defined by aneuploidy or gene rearrangements in the WHO-HAEM4R are retained in the new classifications, though the WHO-HAEM5 uses a shorter nomenclature that does not list cytogenetic changes. The ICC divides the hypodiploid B-ALL/LBL into two subtypes, a low hypodiploid one (32–39 chromosomes), more common in adults, and a near haploid one (24–31 chromosomes), more common in children and associated with poor prognosis and, frequently, with Li-Fraumeni syndrome (germline *TP53* mutation).

The ICC also recognizes two subtypes of B-ALL with *BCR::ABL1*, with possibly different prognosis, one with lymphoid only involvement, and the other with multilineage involvement. The latter entity is not easily distinguishable from CML in lymphoid blast phase and requires demonstration of the *BCR::ABL1* rearrangement in myeloid cells in addition to the lymphoid blasts.

The entity of B-ALL with *BCR::ABL1*-like features /*BCR::ABL1*-like is no longer considered a provisional subtype in the new classifications. The ICC further subtypes it into three subgroups, based on the driver genetic alteration and available targeted therapies: “*ABL1*-class rearranged”,“JAK-STAT activated” and “not otherwise specified”.

#### New entities

Both WHO-HAEM5 and ICC recognize several new genetically-defined B-ALL/LBL entities not included in the WHO-HAEM4R. B-ALL/LBL with *ETV6::RUNX1*-like features (considered provisional by the ICC) is identified by its gene expression profile and usually is driven by fusions or copy number alterations of *ETV6*,* FUS* or *IKZF1*. It may have worse prognosis than B-ALL/LBL with *ETV6::RUNX1.* B-ALL/LBL with *TCF3::HLF* fusion (WHO-HAEM5) /B-ALL/LBL with *HLF* rearrangement (ICC) is a rare entity occurring almost exclusively in children, with very poor prognosis. The most common partner is *TCF3*, but *TCF4* has also been described as an *HLF* fusion partner. B-ALL/LBL “with other defined genetic alterations” is an umbrella category that includes many new entities, some of which are provisional. Most of these entities are recognized by both WHO-HAEM5 and ICC (ALL/LBL with *DUX4*r, with *MEF2Dr*, with *MYC*r, with *NUTM1*r, with *ZNF384*r, with *PAX5* alteration and with PAX5 p.80R), but a few are unique to the ICC classification (ALL/LBL “*CDX2::UBTF*”, ALL/LBL with IKZF1 p.N159Y, ALL/LBL with mutated *ZEB2*/*IGH::CEBPE).*

#### T lymphoblastic leukemia/lymphoma (T-ALL/LBL)

The WHO-HAEM5 classification of T-ALL/LBL is unchanged, with the only distinct variant entity, early T cell precursor (ETP) ALL, identified by immunophenotype.

*BCL11B* activated T-ALL/LBL is a new genetic subtype recognized by the ICC, which encompasses ~ 30% of ETP ALL and is driven mostly by *BCL11B* rearrangements (Table 12).

The WHO-HAEM5 acknowledges the existence of four distinct genetic subgroups of T-ALL/LBL, based on aberrant expression of *TAL* or *LMO*, *TLX1*,* TLX3*, or *HOXA* genes, and also acknowledges the more recent proposal of four additional less common subgroups, also based on aberrant activation of different families of transcription factors [57]. While the WHO-HAEM5 does not recognize these as distinct entities, the ICC lists these eight T-ALL/LBL subgroups as provisional entities, acknowledging limited information is currently available for the four less common subtypes.

**Table 12:** T lymphoblastic leukemia/lymphoma (T-ALL/LBL)

T-ALL entities	Differences between WHO-HAEM5 and ICC
T-lymphoblastic leukemia/lymphoma	Same
Early T-cell precursor lymphoblastic leukemia	Same
Early T-cell precursor ALL with *BCL11B* rearrangement*	Only included in ICC, but not WHO-HAEM5
*TAL1-2* rearrangement*	Only included in ICC, but not WHO-HAEM5
*TLX3* rearrangement*	Only included in ICC, but not WHO-HAEM5
*HOXA* dysregulated *	Only included in ICC, but not WHO-HAEM5
*TLX1* rearrangement*	Only included in ICC, but not WHO-HAEM5
*LMO1-2* rearrangement*	Only included in ICC, but not WHO-HAEM5
*NKX2* rearrangement*	Only included in ICC, but not WHO-HAEM5
*SPI1* rearrangement*	Only included in ICC, but not WHO-HAEM5
*BHLH*, other*	Only included in ICC, but not WHO-HAEM5
Nature killer cell ALL*	Only included in ICC, but not WHO-HAEM5

*Provisional entities in ICC

#### Handling two classifications in diagnosis, therapeutic approach, clinical trials, and research publications

Between 2001 and 2022, the advancement of myeloid neoplasm and acute leukemia classification was sequential, with updates made periodically (in 2008 and 2017) to reflect advancing knowledge. Although some AML clinical trials have even until now retained the antiquated FAB classification for case annotation, in general pathologists, clinicians, researchers, pharmacologic companies, and regulatory authorities such as the FDA have accepted the WHO Blue Books as the single classification to be used as their ‘lingua franca’ for the purposes of diagnosing and studying disease and labelling of specific drugs. Since 2022, this landscape has changed, with the release of two mostly concordant–but often divergent–classification systems. This has created a complex situation on several fronts: (1) Different nomenclature has caused confusion among patients and physicians. (2) Differing diagnostic criteria have resulted in some patients receiving different diagnoses, which may each have unique standards of care. (3) It is unclear how to apply existing drug labelling, which has been largely based on the WHO-HAEM4R, to the new classification systems, or how to label new drug indications in the setting of two classifications with some divergent disease definitions. (4) There is uncertainty as to how researchers and pharmaceutical companies should write inclusion criteria for clinical trials, how to enroll patients in existing trials based on WHO-HAEM4R criteria (many of which have significantly changed in WHO-HAEM5, ICC, or both) and how to stratify patients when studying particular myeloid neoplasms. Practically speaking, diagnosticians, clinicians, and researchers must become familiar with both classifications (Table 13).

**Table 13:** Recommendations on how stakeholders should handle two different classifications of myeloid neoplasms

Individual/Group	Recommended action	Reason
Pathologists diagnosing myeloid neoplasms	Provide both WHO-HAEM5 and ICC diagnoses in pathology reports, whenever there are differences.	Allow facile translation of diagnoses if patients are seen at other institutions or enter trials or research studies.
Researchers reporting studies on myeloid neoplasms	Classify cases according to both WHO-HAEM5 and ICC (or if using one classification, include the other system in supplementary material)	Allow testing of each classification’s criteria for robustness and prognostic relevance; facilitate comparison and meta-analyses of different studies.
Pharmaceutical companies developing drugs to treat myeloid neoplasms	Consider criteria of both classifications when defining the target patient population for a new drug in development	Ensure wider applicability of potential new drugs.
Sponsors and researchers writing clinical trials to study myeloid neoplasms	Write trial inclusion criteria according to both classifications, with careful consideration of the targeted disease.	Promote broader patient enrollment and capture signals that may be better revealed by one classification’s disease definition
Regulatory agencies evaluating new or previously approved drugs that treat myeloid neoplasms	Explicitly include both WHO-HAEM5 and ICC diagnoses in drug labels	Ensure equitable access of patients to new and established drugs, irrespective of which classification their physician or health care system may use
Clinicians treating patients with myeloid neoplasms	Thoughtfully explain different disease names to affected patients and emphasize that disease classification, like selection of therapy, has controversies; consider therapeutic options based on both diagnoses when different	Alleviate patient confusion about their diagnosis; facilitate maximal therapeutic options for patients.

Despite a myriad of publications that have lamented this chaotic situation [58–60], it is important to understand that any classification process cannot be regarded as an absolute truth, but rather represents the efforts of a group of experts to balance scientific evidence with practical considerations of applying diagnostic criteria in the real world. Classifications can harbor errors that warrant correction: for example the purportedly lower-risk ultra-low-blast subgroup of CMML, “CMML-0”, that was introduced in WHO-HAEM4R was subsequently eliminated in both WHO-HAEM5 and ICC due to further evidence showing that CMML-0 in fact has no significant prognostic relevance, as discussed above. These errors underscore the importance of scientific enquiry in both validating and challenging existing classification systems. Although we are now focused on comparing and contrasting the current WHO-HAEM5 and ICC systems, we must look toward the future, at the next classification that will inevitably follow in the next few years. The presence of two ‘competing’ classifications in fact provides an opportunity to engage in scientific testing of both systems, particularly where there are differences. Many such studies testing the differences between WHO-HAEM5 and ICC are already underway or published, and will validate or refute each classification’s criteria in categorizing myeloid diseases [32, 44, 59, 62, 63]. This body of accumulating evidence has the potential to inform a subsequent single classification that will be more accurate, reproducible, and clinically relevant than either the current WHO-HAEM5 or ICC, and most importantly, could serve as a single unified classification accepted by all.

## Data Availability

No datasets were generated or analysed during the current study.
